# No decision about me without me? Shared decision-making in mental healthcare

**DOI:** 10.1192/bjo.2022.75

**Published:** 2022-06-30

**Authors:** Aileen O'Brien

**Affiliations:** Institute of Medical and Biomedical Education, St George's University of London, UK

**Keywords:** Stigma and discrimination, low- and middle-income countries, psychiatry and law, patients, transcultural psychiatry

## Abstract

Is there really ‘no decision about me without me’? This concept of shared decision-making is increasingly supported in the UK National Health Service and is to be welcomed. But the attempt to apply guidelines based on Western physical health settings to all psychiatric patients, across different cultural backgrounds, is problematic. Methodological difficulties when trying to apply the gold standard of randomised controlled trials to the real-life settings of mental health should be considered, especially when many patients with serious mental health problems are excluded, having been deemed to ‘lack capacity’. Should guidelines originating in physical healthcare settings really be applied to mental health ones? Does one size really fit all?

The mantra ‘no decision about me without me’ has found its way into many articles and policy documents on the subject of shared decision-making (SDM).^1^ SDM is defined as ‘a process in which clinicians and patients work together to select tests, treatments, management or support packages, based on clinical evidence and the patient's informed preferences. It involves the provision of evidence-based information about options, outcomes and uncertainties, together with decision support counselling and a system for recording and implementing patients’ informed preferences’.^2^

Is ‘no decision about me without me’ a meaningful statement or a soundbite? In a National Health Service (NHS) Community Mental Health Survey in 2019,^3^ patients were asked if they were involved as much as they wanted to be in decisions about their treatment. and only 51% of people responded ‘yes, definitely’, 38% responded ‘yes, to some extent’ and 12% said ‘no’. Psychiatrists admit that they often are still ‘paternalistic’ when involving patients in decisions,^4^ and describe practical challenges trying to embed SDM in daily clinical practice.

Nevertheless, the UK NHS has made a commitment to SDM as part of universal personalised care, stating that it is ‘appropriate in almost every situation in community, primary and secondary care where a care decision has to be made and that decision is said to be preference sensitive’,^2^ and the National Institute for Health and Care Excellence (NICE) has initiated the first SDM guidelines, for both physical and mental healthcare.^5^

On the surface, it would seem uncontroversial that guidelines about SDM should apply to mental healthcare as much as to physical care, especially in the era of ‘parity of esteem’. However, in their commentary, Zisman-Ilani et al^6^ open a debate about whether one size does, in fact, fit all.

The authors have concerns about the NICE guidelines as they pertain to mental health, describing bias in the evidence investigated and in the lack of attention paid to cultural differences in SDM practice. They argue that important studies were excluded from the evidence because, for example, they did not present an objective primary outcome of SDM, the study design and exclusion of patients lacking the mental capacity to make their own decisions about healthcare. Of course, most clinicians would acknowledge that whatever the assessment of capacity, patients value the opportunity to discuss treatment options with their clinician and have their concerns listened to.

Should the standards of evidence not be the same for mental and physical health guidelines? Zisman-Ilani et al accept that randomised controlled trials are the gold standard, but argue that in this case quasi-experimental designs would be valid. Randomised controlled trials are not always practical to conduct, and may be unrepresentative of populations or conditions found in real-life settings; thus, there has been a growing demand from regulatory bodies to incorporate data from non-randomised studies to cover this ‘gap’.^7^ There is a need for more debate about the balance between real-life pragmatism and traditional standards of evidence in this patient group.

The exclusion of patients without ‘capacity’ in research generally is ethically contentious.^8^ It arguably could exclude a group of people who may well express a view about their treatment even if their capacity is assessed as being fluctuating or borderline. If, as the authors point out, patients without ‘capacity’ are excluded from trials, how do we apply interventions to our patients when we know that in real-life settings, capacity is so much more complex than a binary assessment, that it fluctuates over time and is context specific?^9^ It may be that evidence tested on those assessed as having capacity can be extended to those without, but we just do not know if that is the case.

This point about capacity is of particular relevance in England and Wales, given the recent review of the Mental Health Act 1983 and plans to introduce statutory support for mental health advance decision-making in the form of advance choice documents.^10^ It is a concern that the literature on advance decision-making was not included in the SDM NICE review, when advance choice documents could potentially be a tool to enable people with fluctuating capacity to take part in SDM.

It is understandable that policy makers would wish to avoid treating psychiatry as ‘different’, hence sticking to the same criteria as for general medicine when establishing guidelines. It could be suggested that this is in an effort to reduce stigma, the implication being that the same standards of evidence should apply to both. But it is arguably disingenuous to suggest that guidelines designed for diabetes can be used in schizophrenia without any thought to some of the fundamental differences between the conditions, for all the reasons that Zisman-Ilani et al argue. Similarly, it is disingenuous to assume protocols originating in physical healthcare settings can automatically be applied to mental health ones. Some would argue that the distinction between mental and physical healthcare is arbitrary and best viewed on a continuum. However, the law in the UK has developed along a medical/psychiatric division and there are differences; as Owen et al argue, capacity fluctuates more in mental health conditions, with the potential for much greater changes in a decision than one would routinely find in people making advance decisions in physical healthcare. There are also ethical challenges if a patient's advance decision declines life-saving treatment for a mental health problem, or if the refusal of treatment presents a risk to others.^11^

The argument that the same evidential rigour should be applied to psychiatric guidelines as for those with physical health conditions has strengths, but the criticism regarding the guidelines’ lack of attention to cultural differences seems more difficult to defend. The challenges of navigating different healthcare structures and varying sociocultural beliefs about medicine should not be underestimated, but the implication that guidelines based on English-language research alone is applicable to all cultures feels problematic.

If SDM is something we should aspire to in psychiatry, and it would seem an unusual position to argue otherwise, there needs to be an understanding that although psychiatry deserves parity of esteem, there are real-world differences to general physical healthcare. For these guidelines, and in similar initiatives, we need more research in real-life psychiatry settings and non-Western cultures before we try to mould those designed for physical health conditions in the West and assume without modification that they will be the right fit for the rest.
